# Conflict and crime. Restorative justice in Italy

**DOI:** 10.3389/fsoc.2023.1175291

**Published:** 2023-08-28

**Authors:** Giovanna Palermo

**Affiliations:** Department of Psychology, University of Campania Luigi Vanvitelli, Caserta, Italy

**Keywords:** conflict, crime, victim, restorative justice, criminal justice, sociology of law

## Abstract

The article presents a socio-legal analysis of the Legislative Decree (Lgs.D.) of 10 October 2022, n.150, implementing the law of 27 September 2021, n.134 (so-called “Cartabia reform”), which provides for the introduction in Italy of a comprehensive framework regulation of restorative justice. The central theme of this paper is precisely the conflict connected to a crime, the offender-victim relationship and restorative justice in the light of the aforementioned decree. As Luhmann observes, the omnipresence of conflicts in society is self-evident. Even the crime produces a conflict or, at times, is the extreme expression of a pre-existing and sometimes latent conflict. The problem that arises is, therefore, that of the control and management of conflicts. These are usually managed by the law, but, given its limitations, it is increasingly necessary to encourage the use of restorative justice programs. On an interpersonal level, conflict triggers a negative mechanism of hostility and disavowal of the other, which either wraps itself in an explosive vortex, or deviates, becoming the pretext for abandoning the only form of communication that conflict makes possible, as Luhmann observes, the one based on “no”. The Italian regulatory intervention certainly appears relevant, albeit with some critical issues, and to be kept under observation for future application developments.

## 1. Introduction

There is a close relationship between crime and conflict, between criminally relevant conduct and that relational dimension, in which there is a divergence between two or more people on apparently irreconcilable positions and interests, with respect to which each contender wants to maintain their position.

The conflict can be the premise of the crime, its original matrix, but it can also emerge as a consequence, as an effect of the criminal event.

There is therefore a close link between crime and conflict. From the analysis of the conflict between offender and victim emerges the residual role that the law has always recognized to the victim.

In fact, since the middle of the last century there have been criminal and prison policy choices that have favored that attitude of “victimization of the offender and scotomization of the victim” (Ponti, 1995). For years, the rehabilitation model has mainly focused on the offender, denying the victim. The inability of the penal system to achieve general and special prevention objectives and to satisfy the crime victim has weakened the sense of trust in criminal law and institutions in general (Bazemore and Walgrave, 1999; Bazemore and Schiff, 2002/2015; von Hirsch et al., 2003; Gavrielides, 2007; Dünkel et al., 2015; Brennan and Johnstone, 2018; Vanfraechten and Aertsen, 2018).

Indeed, the different social reactions to the crime, from the request for punishment of the offender to the request for defense and security of citizens, from the isolation of the perpetrator of the crime to the idea of his necessary re-education, no longer provide a guarantee for the re-composition of the rift personal and social. In the face of these failures and also as a function of deflating the judicial burden, in Europe, since the 1970s, various pilot projects have followed one another (Norway, Finland, Austria, England) and attempts at legislative reforms on restorative justice (Braithwaite, 1989; D'Amato, 2018; Cartabia and Ceretti, 2020).

In Italy, recourse to restorative justice has always been limited to isolated experiments, due both to the absence of a comprehensive framework regulation and to the criminal-centric approach of the Italian system.

Today, Legislative Decree No. 150 of 10 October 2022 drew attention to the need to include restorative justice programs in our penal system, for a comprehensive management of the victim-offender conflict.

This legislation which will lead to the institutionalization of restorative justice processes, with all the risks of distorting them, putting at risk their informal and voluntary nature and their alternative language, certainly appears to be relevant and to be kept under observation for future application developments.

## 2. Conflict of the crime, scotomization of the victim and limits of the penal system

Conflict is a natural expression of living together and is a relational modality that characterizes all social systems. As an interaction between incompatibilities, it appears, as Luhmann (1984) observes, whenever a contradiction is communicated. Once it has arisen, the conflict stabilizes as a system that integrates and aggregates and its destructive force is not produced within it, but “it lies in a relationship with the system in which the conflict found an occasion and outlet perhaps in a relationship with a neighbor, in a marriage or family, in a political party, at work, in international relations, and so forth” (Luhmann, 1984, p. 391). In this sense, the conflict system will manifest its “parasitism”, tending “to draw the host system into conflict to the extent that all attention and all resources are claimed for the conflict” (Luhmann, 1984, p. 391).

From Luhmann's point of view, conflict is therefore not necessarily dysfunctional to the social system, on the contrary it performs an irreplaceable function of indicator of dysfunctions within it. On the other hand, it is the forms of destructive management that have a negative value and must therefore be controlled and oriented. I think this approach is the premise that can be shared to support the usefulness of restorative justice interventions.

Conflict management has always been entrusted to the law, but the penal system has a criminal-centric approach, as it is concerned exclusively with giving guarantees to the offender.

So for years the role of the victim has been considered exclusively as functional to the initiation and continuation of the criminal process, the real protagonists are the criminal and the State in a diatribe between the need to exercise the right-duty of the latter to “punish” and the need to guarantee the alleged offender.

Moreover, the judicial system does not have the main function of responding to the needs of the victim: it has its own concepts of justice, truth, damage and follows other imperatives.

Yet on the political level we have been witnessing for years a season of protagonism of the victim, which is favoring the proliferation of regulatory interventions on restorative justice.

Already in the early 70s of the last century England promoted a significant law on public compensation for victims of crimes of violence, but also Canada, some states of the USA, Australia, as well as the Scandinavian countries, began to affirm “the theme of the victim as the one who bears the social cost of a collective risk, of a risk of the metropolitan organization” (Pavarini, 2001, p. 9).

The same critical movements of criminal law have gone as far as extreme positions, such as that of abolitionism, openly in favor of the disappearance of the institutions of the penal system and of the assignment to civil society of the task of regulating individual conflicts (Christie, 1977).

In reality, “what is still unknown is the perspective of the victim: his way of seeing and thinking about the things of justice” (Palermo, 2016, p. 66).

The judicial process is a process “of” and “on” manifest behaviors and does not give space to logics other than opposition. The logic of reciprocal attacks that characterizes the conflict more often than not does not subside even with a possible decision in court. In many cases, indeed, it is accentuated, both by the conflicting party who “won” and by the one who “lost” and who wants to make up for it in some way.

Yet, the trial before the judicial authority is still seen as the only and irreplaceable tool for restoring the violated interests and social peace despite the obvious limitations. These are limits dictated by the fact that conflicts involve more complex dynamics, which go beyond manifest behavior and also and above all involve subjective perceptions and experiences that remain hidden (Galtung, 1969).

An alternative method to guarantee management of the conflict connected to the crime in its complexity and to restore a role to the victim in the criminal process is represented by restorative justice programs. In fact, they highlight the need to break and overcome the conflicting logic, through the rediscovery of other communication levels, avoiding pathological interpretations of the conflicting story and, instead, reworking the critical event in terms of relational reorganization.

## 3. The comprehensive framework regulation of restorative justice in Italy

Restorative justice is “other” justice, which ignores the definitions and roles that the law crystallizes in the criminal process and in the application of the penalty. It is a justice that abandons the winners-losers logic and deals with relational conflict.

It does not consider the crime in the abstract, as an element that disturbs the social balance, as an offense against society, which requires punishment, but as an expression of a specific micro-conflict, which causes suffering, deprivation and which requires the activation of forms of communication and, possibly, reconciliation and reparation, also with a view to strengthening the sense of belonging and collective security.

In this perspective, it is increasingly necessary to ensure the relational nature of restorative justice processes, voluntary access, providing for the need for free, informed and revocable consent at any time, without negative consequences on the pending criminal trial, ensuring voluntariness also with reference to the assumption of any commitments that may arise.

To date, the Italian legal system has recognized two areas of intervention for the victim: probative solicitation and control and impulse of the criminal action, not giving due importance to possible requests for reparation.

The Italian penal system has a decidedly “pro-criminal” vision, neglecting the victim and the entire parental, environmental and social entourage.

Over the years, however, there have been attempts to experiment with mediation processes in the criminal trial, especially in that against minors (Scivoletto, 2022).

Despite these implementations on an experimental basis, in Italy restorative justice is practically unknown to a large part of the citizenry and to a large part of legal practitioners.

Not so much the results of these implementations, therefore, as the need to take charge of the management of the conflict between victim and offender, to recognize the victim a central role in the criminal process and also a reparation, as well as to promote the responsibility and resocialization of the offender were the political and juridical reasons that led the legislator to implement the recourse to restorative justice.

For this a profound and substantial reform was carried out by Legislative Decree No. 150/2022, which provided for the introduction of a comprehensive framework regulation of restorative justice, theoretically applicable to any type of crime.

This justice “enables the victim of the crime, the person named as the perpetrator of the offense and other subjects belonging to the community to participate freely, in a consensual, active and voluntary way, in the resolution of the issues deriving from the crime, with the help of an impartial third party, adequately trained, called mediator”.

The reference for the first time to the community responds to an enlightened vision of restorative justice, which should stop being a “private” matter between the offender and the victim, to become of social interest. Restorative justice no longer just as a tool for managing internal conflict, but as a means that produces its effects on the whole community.

The outcome of this path is an eventual agreement, an expression of mutual recognition and of the possibility of reconstructing the relationship between the participants, aimed at the symbolic or material reparation of the offense.

The reform clearly establishes that restorative justice programs must aim to promote the recognition of the victim of the crime, the accountability of the offender and the re-establishment of ties to the community, but it will be necessary to see what will be achieved on a substantial and not a purely formal level.

Access to restorative justice programs, in addition to being free, is permitted at every stage and level of the criminal proceeding, as well as in the execution phase of the sentence or even after its execution.

The mediators, who must be at least two, and the staff of the restorative justice centers are bound to the confidentiality of activities, deeds, statements, information, which are unusable in criminal proceedings. The meetings between the subjects involved must be held in suitable spaces and places to ensure confidentiality and independence.

Legislative Decree No. 150/2022 provides among the restorative justice programs in addition to mediation between perpetrator-victim-community, also the reparative dialogue and any other perpetrator-victim dialogue program.

At the end of the chosen program, a report is sent to the proceeding judicial authority, drawn up by the mediators and containing the description of the activities carried out and the results achieved, or the failure to carry out the program, the interruption of the same or the failure to achieve a remedial result. However, the latter hypothesis cannot produce unfavorable effects on the person indicated as the perpetrator of the offense.

The proceeding authority will evaluate the implementation of the program and any remedial outcome for the determinations of its competence, also for the purpose of assessing the seriousness of the crime.

Lastly, the new legislation made inevitable changes to the penal code, introducing the provision of the tacit remission of the lawsuit and a common mitigating circumstance in the event that the alleged offender has participated in a restorative justice program with the victim of the crime, concluded with a restorative result. A similar circumstance is also relevant for the purposes of applying the conditional suspension of the so-called ≪short≫ or ≪special≫.

Furthermore, some changes were also inevitable at a procedural level, to insert the reference to the “power to access restorative justice programs” in the various provisions governing the information rights of the suspect/accused and the offended person.

Legislative Decree 150/2022 has today also amended some rules relating to juvenile criminal proceedings and the execution phase to include explicit reference to restorative justice programs. In particular, the modification concerns the institution of the suspension of the process and probation. This institute had already been used in the past to allow the inclusion of mediation, at the discretion of the judge. Today the judge, with the order suspending the trial, can not only give the accused prescriptions aimed at repairing the consequences of the crime and promoting conciliation with the person offended by the crime, but also invite him or her to participate in a restorative justice program.

## 4. Conclusions

The reform represents the first explicit attempt to regulate restorative justice in detail in Italy, overcoming the previous attempts of reduced, sectoral and partial interventions that have taken place in the past. However, we can already grasp some critical issues.

In particular, the prediction of the applicability of restorative justice programs in each phase of the process and also in the execution phase of the sentence poses a problem of opportunity.

In fact, I believe that the preliminary investigation phase of the criminal trial is the best time to launch restorative justice programs, since it is free from the prejudices and distrust that occur in the subsequent phase and because the objective of these programs should be also to facilitate the release of the offender from the criminal circuit as soon as possible. In the execution phase of the sentence, these programs would no longer be able to achieve their goals, first of all, the recognition by the offender of the person-victim.

In fact, it does not seem to be in the interest of the offender and, perhaps not even of the victim, to start a communicative-relational process at a time when the criminal act is temporally far away and criminally already defined.

The decree ends up distorting the essence of restorative justice programs, as it jeopardizes the informal and voluntary nature of restorative justice and its alternative language, which could irreparably be included and homogenized with that of the criminal justice system.

The decree also missed the opportunity to show the effective will to give the victim back a central role in the process. In fact, it should have provided for the creation of assistance centers for victims alongside all criminal-centric bodies and institutions. These centers, also physically inserted in each Court, could have had the fundamental task of assisting the victims, protecting them and, symbolically, would have sanctioned the visible presence of the victims in the places historically dedicated to the perpetrators of the crimes, favoring the overcoming of the criminal vision central to our penal system.

The reform, on the other hand, is limited to providing for the establishment of Centers for restorative justice in local authorities, with the task of ensuring essential and uniform levels of provision of services for restorative justice. These centers, at local authorities, have the exclusive purpose of optimizing the implementation of restorative justice programs from an organizational point of view and therefore end up performing a function in the interest of service management, in favor of all the subjects involved.

On the other hand, a strong sign of the overcoming of the criminal-centric approach of criminal system would have been the creation, parallel to and in addition to them, of centers in each court with the specific function of protecting and assisting victims.

In any case, this regulatory intervention could promote a cultural change, facilitating the transition from the rehabilitative, criminal-centric model to a “relational justice” model, in which the focus shifts not only to the offender, but also to the victim and the society.

This new model represents much more than the simple application of a conflict management technique, because it takes the form of a process capable of producing a new sociality.

If these are the critical issues and the possible application scenarios, we cannot ignore the fact that the objective of the legislator appears essentially and mainly to favor a reduction of the burden of proceedings, with a view to improving the effectiveness of the criminal justice system.

In addition, the path for an effective and efficient reorganization of the penal system also from the point of view of restorative justice is to be “built” and will inevitably collide with the power of the law to take over the management of the conflict connected with the crimes and to inflicting the penalty as a means of social discipline, in the name of a moral and retributive function of the penalty.

Therefore, if the Italian legislator^1^ has formally opened the doors to restorative justice, it will be necessary to see how effectively the law will be willing to lose the monopoly in the management of crime and the power of differential control of illegalities.

## Data availability statement

The original contributions presented in the study are included in the article/supplementary material, further inquiries can be directed to the author.

## Author contributions

The author confirms being the sole contributor of this work and has approved it for publication.

## Footnotes

^1^The entry into force of the provisions on restorative justice is scheduled for 30 June 2023.
